# Intelligent Monitoring System with Privacy Preservation Based on Edge AI

**DOI:** 10.3390/mi14091749

**Published:** 2023-09-07

**Authors:** Soohee Kim, Joungmin Park, Youngwoo Jeong, Seung Eun Lee

**Affiliations:** Department of Electronic Engineering, Seoul National University of Science and Technology, Seoul 01811, Republic of Korea; kimsoohee@seoultech.ac.kr (S.K.); parkjoungmin@seoultech.ac.kr (J.P.); jeongyoungwoo@seoultech.ac.kr (Y.J.)

**Keywords:** SoC, edge AI, intelligent monitoring system, privacy preservation

## Abstract

Currently, the trend of elderly people living alone is rising due to rapid aging and shifts in family structures. Accordingly, the efficient implementation and management of monitoring systems tailored for elderly people living alone have become paramount. Monitoring systems are generally implemented based on multiple sensors, and the collected data are processed on a server to provide monitoring services to users. Due to the use of multiple sensors and a reliance on servers, there are limitations to economical maintenance and a risk of highly personal information being leaked. In this paper, we propose an intelligent monitoring system with privacy preservation based on edge AI. The proposed system achieves cost competitiveness and ensures high security by blocking communication between the camera module and the server with an edge AI module. Additionally, applying edge computing technology allows for the efficient processing of data traffic. The edge AI module was designed with Verilog HDL and was implemented on a field-programmable gate array (FPGA). Through experiments conducted on 6144 frames, we achieved 95.34% accuracy. Synthesis results in a 180 nm CMOS technology indicated a gate count of 1516 K and a power consumption of 344.44 mW.

## 1. Introduction

Due to the declining birth rates and rising life expectancy, the process of demographic aging is advancing [1]. According to statistics in South Korea, the proportion of the elderly population will steadily increase from 16.5% in 2021 to 43.9% by 2060. This shift toward an aging society is an issue that most advanced countries are facing, not only Korea [2,3]. In the future, dealing with a large aging population with poor economic capabilities will pose significant challenges globally. The elderly need continuous attention and assistance, as they often face physical vulnerabilities with chronic health conditions, as well as social and economic limitations [4]. Within the elderly demographic, those living alone are a particularly high-risk group. Elderly individuals living with their families can expect to receive support and care from their family members, whereas those who live alone do not have the same expectations. In this societal context, media reports have increasingly highlighted cases of lonely death among the elderly, and social interest has increased.

Lonely death, as stipulated by the Korean constitution for prevention and management, is defined as the death of a person who lives alone, isolated from family, relatives, and others, and is discovered only after some time has passed due to causes like suicide or illness [2]. Recognizing this specific form of death as a word and a social problem indicates that lonely death is viewed as a violation of human dignity. This leads to a social consensus built upon the belief that it is disrespectful to the deceased and even results in legislation to prevent lonely deaths. The issue of lonely death is expected to cause serious social problems in demographic structures, not only in the elderly population but also single-person households. To cope with these problems, the Ministry of Health and Welfare supports elderly care services, such as living assistance, visits, and phone calls [5]. However, due to the labor-intensive nature of these services, which require direct human involvement, the supply of caregivers is unlikely to meet the growing demand, resulting in higher care costs and a decrease in the quality of life [3]. The Ministry of Health and Welfare recognized the previously mentioned problems and provided a monitoring system by applying the Internet of Things (IoT) technology. However, despite technological advancements, equipment older than five years has been used due to difficulties in obtaining parts [2]. Furthermore, as the number of clients increases, there have been issues with stable service provisions due to device malfunctions and data transmission errors [6,7,8]. Existing monitoring systems utilize multiple contact/non-contact sensors to observe the states of elderly people who live alone and implement cloud computing approaches, where collected information is transmitted to a database server for processing [9]. In wearable or IoT sensor-based monitoring systems, user convenience is limited [1,10,11]. In addition, implementing multiple sensor-based systems to manage all aspects of daily life is not economically beneficial [4]. Also, IoT sensors are sensitive to changes in detection environments, such as contact with the target or transitions in temperature and humidity, which may result in inaccurate results [12]. The most important aspect is that, in cloud computing, where a server-based approach is used, user information is continuously exposed to the server, and if it is hacked, it can lead to severe breaches of personal information [13,14,15].

Given these facts, a monitoring system must be designed to reflect the characteristics of elderly people living alone. First, elderly individuals living alone may prefer to sustain their lives in their chosen spaces rather than in healthcare facilities [4,16]. Considering these characteristics, monitoring devices should be designed for easy installation in any environment without costly remodeling. Second, elderly people living alone are vulnerable to economic insecurity due to retirement or health issues [4]. Therefore, monitoring systems should be manufactured to ensure maintenance, efficiency, and affordability, taking income levels into account. Third, the system should not adversely affect the quality of life of elderly people living alone; it should ensure convenience in their daily activities. Fourth, designs that consider data leakage prevention are essential, since monitoring systems deal with sensitive information about the subjects being observed [15]. Lastly, since the system operates 24/7 and responds to increasing demands, it should have a structure that manages the increase in data without overloading the network, and provides services that operate efficiently with minimal power consumption [16,17].

In this paper, we propose an intelligent monitoring system with privacy preservation based on edge AI. The proposed system is an embedded monitoring system based on a single sensor, utilizing a robust camera module that is resilient to external disturbances, such as changes in the surrounding environment. By employing vision-based recognition techniques using cameras in the embedded system, the presence and movements of elderly individuals living alone are detected, and only the identified information regarding their status is transmitted to the server. In an embedded system, devices face difficulties in performing artificial intelligence algorithms due to their limited sizes and low-power characteristics. To address this issue, we constructed a system that includes a dedicated processor specifically designed for the *k*-NN (k-nearest neighbor) algorithm, enabling low-power and high-efficiency computations. By applying edge computing technology, we eliminated the process of transmitting a significant portion of input data from the client to the server, a process that was used to cause network congestion. Moreover, the complex computations that were previously performed on the server are now distributed and executed by the client, resulting in a reduction in performance requirements for the server [18,19,20]. Additionally, to maintain a low-power design, we incorporated a wake-up function that initiates AI algorithm computations solely when an individual is detected.

The contributions of this paper are as follows. We propose a security architecture that uses edge computing technology and an edge AI module for an embedded intelligent monitoring system. The intelligent monitoring device includes an edge AI module. We enabled low-power, high-efficiency computations by constructing systems with dedicated processors specifically designed for AI algorithm operations [21,22,23,24,25]. The accuracy of the vision-based monitoring system was analyzed to demonstrate the feasibility of the system. In addition, edge computing technology was applied to handle the increasing communication workload in the system, and the edge AI module blocks communication between the camera module and the server, increasing the security and prevention of personal information leakage [14,15,17,26,27].

The remainder of this paper is organized as follows. In Section 2, we discuss related works. This includes studies on existing monitoring systems for elderly people living alone; we describe the trends in research focused on lightweight AI implementation in embedded systems and in designing accelerators that harness lightweight AI. Section 3 introduces the structure of the proposed vision-based intelligent monitoring system. Section 4 describes the algorithm of the proposed system. This section explains the overall flow of the intelligent monitoring system, including the image preprocessing sequence and the learning/inference algorithm of the edge AI module. In Section 5, the experiments and results of the proposed system are presented, including an explanation of the verification results through the FPGA implementation. Finally, Section 6 concludes the paper.

## 2. Related Work

As machine learning, AI, sensing technology, and computer vision rapidly advance, research on sensing and modeling human activity is actively underway [2,4,28,29,30,31,32,33]. Due to these studies, applications utilizing technology for activity recognition (AR) are emerging. Due to societal aging, there has been an increasing demand for monitoring services targeting elderly people living alone. The research focused on monitoring the activities of daily living (ADL) of elderly people living alone—using various IoT technologies and AI-based AR analysis technologies—is actively being conducted. In Table 1, an analysis of related works is presented. ADL has been used as an indicator in elderly research to evaluate daily activities, such as eating, dressing, and using the bathroom [15]. Previous studies have been conducted by applying numerous IoT sensors and complex AI algorithms on servers to identify and classify ADL and gain insight into household residential behaviors [10].

In [2], the authors looked into platforms based on IoT sensors for elderly people living alone. A camera, a passive infrared (PIR) sensor, and a sound sensor were installed on Raspberry Pi to detect the movements and sounds of elderly people living alone, and the modules were controlled with Python. They proposed an improved speed-up robust feature (SURF) image detection algorithm for motion detection, which utilizes the slope between matched feature points in consecutive frames. They also suggested the SSC (silverlinker situation classification) algorithm to classify situations into ‘safe’, ‘caution’, and ‘danger’ categories, based on the detected data, and they utilized the situation classification function. The system employed a server and database for communicating between Raspberry Pi and the mobile application. In [28], multiple acoustic sensors were placed in different locations around the home to automatically monitor the daily activities of the elderly, such as dishwashing, meal preparation, and eating. This study used the sound obtained from a wireless acoustic sensor network (WASN) installed in a home environment to extract the mel-frequency cepstral (MFCC) coefficient, which is a feature extraction approach that is commonly used in speech and speaker recognition applications, and performs ADL classification with support vector machines (SVMs). In [29], multiple ultrasonic sensors were installed in various locations within a home, such as the ceiling, to perform distance measurements between the subjects and sensors. This enabled the system to detect whether individuals were standing, sitting, or have fallen; it also detected changes in their movements. To detect whether a person was standing, sitting, or had fallen, SVM with linear kernel, *k*-NN, and decision tree techniques were applied to the data collected by ultrasonic sensors. This system achieved activity detection accuracy of 90% in laboratory settings. In [30], binary sensors, such as PIR, as well as door sensors, were used to detect and classify elderly activities, and the binary data were converted into binary activity images. Activity images were used as training and testing data for deep convolutional neural network (DCNN) classifiers; classifiers were evaluated via 10-fold cross-validation. Four ADLs—bed-to-toilet movement, eating, preparation of meals, and relaxing—were detected, and the DCNN classifier presented an average accuracy of 99.36%. In [31], the authors employed a magnetic contact sensor to evaluate the user’s position, a PIR sensor to collect motion information, a force/pressure sensor to obtain primary information about the user’s position, and an electrical usage sensor to recognize the usage of electrical appliances used by the user. The data collected from the sensors were filtered; spikes produced by the electrical usage sensor of the personal computer were filtered using the median filter. After filtering, samples from the binary sensor network deployed in the environment were processed by the “where is” (WHIZ) algorithm, providing information about the locations of the elderly. The proposed system detected ADLs, such as lunch/dinner, resting/PC/TV, sleeping, and hygiene, and achieved a sensitivity of 81%.

**Table 1:** Analysis of related work.

Source	Sensor	Proposed Approach	Outputs	Pros	Cons
[2] Han-Sol et al.	Camera sensor, PIR sensor, sound sensor	(1) Enhances SURF algorithm: Detects motion between consecutive frames by using the gradients of matched key points.	Detects motion and sound.	It allows precise motion detection.	Unnecessary motions are detected, leading to resource wastage.
[28] Vuegen et al.	27 (Number of microphones)	(1) Mel-frequency cepstral coefficient (MFCC) approach: Feature extraction from acoustic sensor data performed (2) Support vector machine (SVM): ADL classification.	Detects teeth-brushing, dishwashing, dressing, eating, food preparation, table setting, showering, sleeping, toileting, and hand-washing.	Requires only sound sensing technology. Provide rich information	Many sensors are required. Does not preserve privacy.
[29] Ghosh et al.	5 (Number of ultrasonic sensors)	(1) Support vector machine (SVM) with linear kernel, K-nearest neighbor (KNN), and decision tree techniques: Used on ultrasonic sensors data	Detects standing, sitting, and falling.	Utilizes lightweight algorithms.	Ultrasonic sensors are vulnerable to disturbances.
[30] Gochoo et al.	PIR, reed switch	(1) The annotated binary data are converted into binary activity images for ADLs. (2) Deep convolutional neural network (DCNN) classifier: Activity images used for training and testing. (3) Classifiers evaluated with the 10-fold cross-validation method.	Detects four ADLs: bed-to-toilet movement, eating, meal preparation, and relaxing.	Performs highly accurate deep learning algorithm computations.	It results in a system that is too heavy for certain environments.
[31] Barsocchi et al.	PIR, Reed switch, pressure sensor, electrical power sensors	(1) Magnetic contacts and power usage sensors: Detects status changes. (2) Median filter: Applied to the spikes produced by the power usage sensor of the personal computer. (3) Room-level localization algorithm “where is” (WHIZ): Detects the locations of the elderly.	Detects ADLs such as lunch/dinner, resting/PC/TV, sleeping, and hygiene.	Employs various sensors to sense real-life situations more closely.	It necessitates the use of many sensors.

In the proposed system of this study, an AI accelerator applicable to embedded systems is included to implement the intelligence of the monitoring system. To apply AI to embedded systems, low-power, high-efficiency designs that consider the limited power supply are essential. Lightweight research on AI algorithms has been conducted to implement AI in embedded systems, as well as research to enhance computational performance through hardware designs dedicated to the operation of such algorithms [34,35,36].

A low-power deep neural network (DNN) accelerator for mobile machine learning applications based on embedded systems is proposed in [34]. The designed DNN accelerator is lightweight as it applies weight pruning and non-linear quantization; after compressing the weights using Huffman encoding, it performs DNN operations on-chip by storing them in the embedded resistive RAM (RRAM) without using external memory. A neural CPU architecture for embedded AI based on the RISC-V platform is presented in [35]. The heterogeneous architecture includes a general-purpose processor and AI accelerator, where the CPU performs data preprocessing and the processed data are input into the AI accelerator for computation. However, the resource usage efficiency is reduced due to the division of the workload between the CPU and AI accelerator. To address this issue, the binarized neural network (BNN) layer was mapped onto the pipeline stage structure of RISC-V, enabling the CPU to share hardware resources with the AI accelerator during preprocessing and computations, resulting in a 35% reduction in the design area and 12% improvement in power efficiency. A reconfigurable lightweight convolutional neural network (CNN) accelerator based on FPGA is suggested in [36]. Although various vision tasks based on CNNs are implemented on embedded platforms, the resource usage efficiency is reduced when performing these operations using a general-purpose CNN engine. To resolve this problem, a CNN accelerator architecture capable of reconfiguring the convolution layer and optimizing data flow was suggested.

## 3. System Architecture

**Figure 1:**
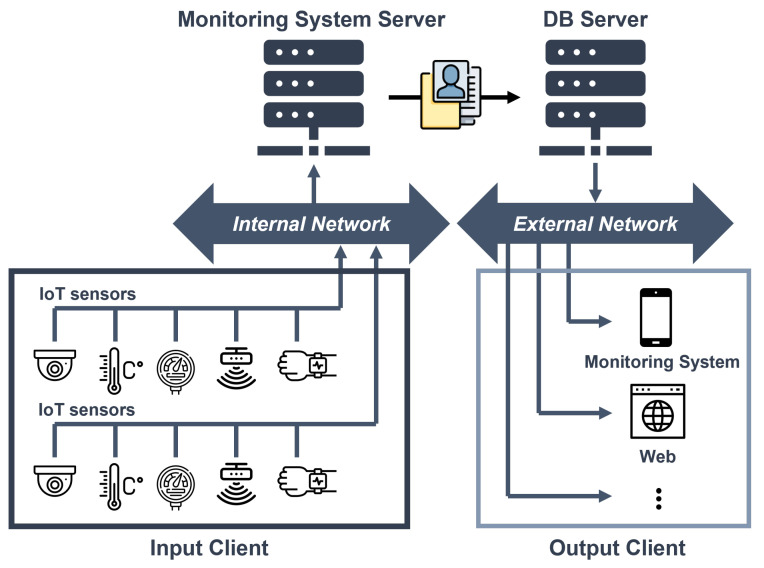
Existing monitoring system.

The proposed elderly monitoring system was designed by employing a dedicated AI processor to meet the requirements of the elderly monitoring system application. It primarily utilizes vision-based recognition techniques to determine the presence and movement of individuals. Figure 1 illustrates the existing monitoring system. The client, which includes various sensors, transmits the sensor measurements of the observed subject to the server. The server then applies algorithms such as image processing and data processing to determine the person’s status. The recognition results are sent to the database server and displayed on an external monitoring system. However, this process causes a significant load on the server due to the data transmission and computation from numerous sensors, requiring high-performance servers.

**Figure 2:**
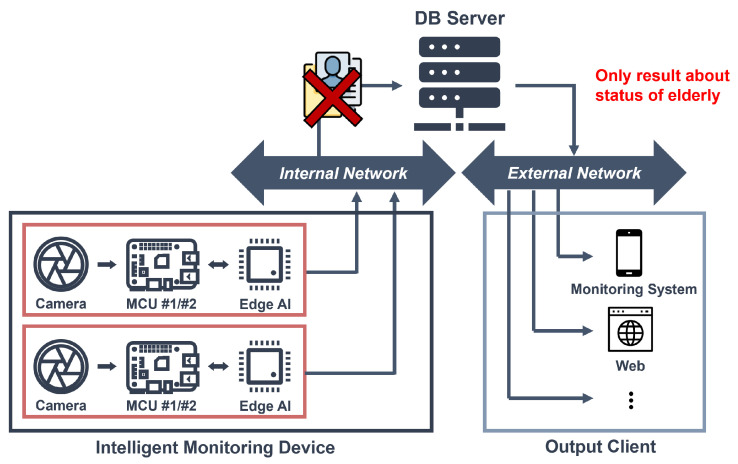
Vision-based intelligent monitoring system.

Figure 2 illustrates the vision-based intelligent monitoring system that we propose. An intelligent monitoring system that is capable of autonomously determining the presence and movements of individuals is proposed to reduce the server’s burden related to image data transmission and processing and prevent personal information leakage. In the proposed system, an edge AI module assesses human movements and communicates the recognition results to the MCU. The processor generates danger detection signals based on the recognition results and sends them to the server, where external monitoring systems receive alerts for risk detection. The server only receives the individual’s status results from the intelligent monitoring device, ensuring fundamental prevention of personal image data leakage and reducing the server’s performance requirements.

### 3.1. Intelligent Monitoring Device

**Figure 3:**
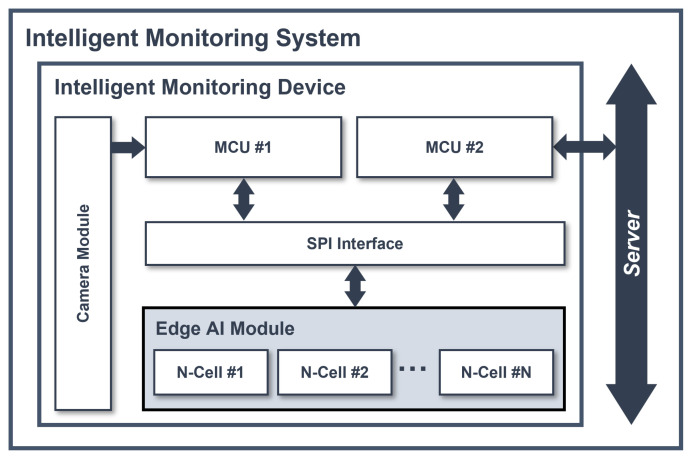
Architecture of an intelligent monitoring system.

Figure 3 presents the architecture of an intelligent monitoring system, which detects and classifies the ADL of elderly people. An intelligent monitoring device consists of one camera module, two microcontroller units (MCUs), and one edge AI module. MCU no. 1 receives the image of the elderly individuals from the camera module, preprocesses the image utilizing MediaPipe and the image preprocessing algorithm, and converts it into 128 byte vector data points, compatible with the input of the edge AI module. The preprocessed data are then passed as input to the edge AI module, along with inference and result commands sent via the serial peripheral interface (SPI). MCU no. 2 receives the category value derived from the result after the edge AI module completes the inference task. Based on this category value, MCU no. 2 generates an abnormal detection signal. If the category value has not changed for a certain period of time, MCU no. 2 determines that the elderly person living alone has passed away, and transmits this signal to the server. Looking at the image data flow, the edge AI module is placed between MCU no. 1 and MCU no. 2, preventing the raw data from being transmitted to the server. With this architecture, the proposed edge AI device can prevent the leakage of personal information.

### 3.2. Edge AI Module

**Figure 4:**
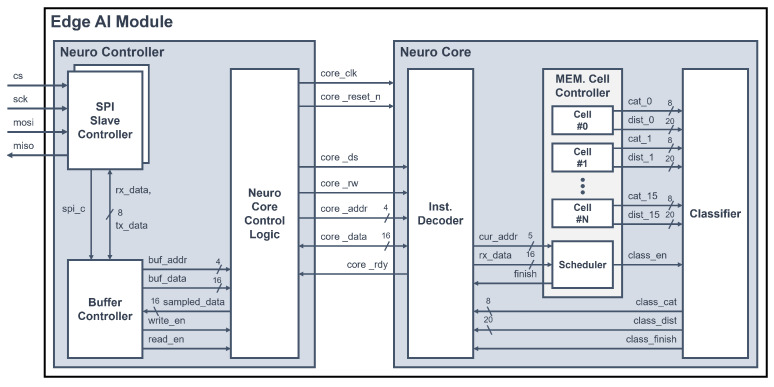
Architecture of the edge AI module.

The proposed system includes an edge AI module that performs AI computations on its own to enhance computational performance, a key feature in recognizing the state of an elderly person living alone. Considering the power and area design goals for embedded systems, the proposed edge AI module is designed to specifically perform a lightweight AI algorithm, the *k*-NN algorithm [37]. The *k*-NN algorithm is a supervised learning algorithm that performs learning and inference based on distance calculation. It stores training data and their corresponding labels, which represent the data categories. When data for inference are transmitted, the distances between the learning data and the transmitted data are calculated, and the calculated distance values are compared. By comparing the calculated distance values with the labels of the most similar training data for the k calculated distances, the labels of the most similar training data are outputted to classify the inference data. Figure 4 shows the architecture of the edge AI module [37]. The edge AI module consists of a neuro controller and a neuro core. The neuro controller receives commands and data through the SPI interface. Then, it interprets the received data to generate and transmit signals that operate the neuro core. The neuro core inputs the training data to the memory cell or receives the input data, then calculates the distance between the input data and the data in the memory cell to output the inference result.

#### 3.2.1. Neuro Controller

The neuro controller is composed of two SPI slave controllers to communicate with two MCUs—a buffer controller and a neuro core control logic. When the SPI slave controller receives data from external sources, it transfers the received 8-bit data (rx-data) to the buffer controller and simultaneously receives 8-bit data (tx-data) to be transmitted from the buffer controller to send it out. The buffer controller interprets the data received through the SPI slave controller and generates a 4-bit memory address, 16-bit data, writes the enable (write_en) signal, and reads the enable (read_en) signal to transmit to the neuro core control logic. In addition, the buffer controller receives the 16-bit data (sampled_data) produced by interpreting the resulting data of the neuro core from the neuro core control logic and generates the data to be transmitted (tx-data). The neuro core control logic generates the operation signals, commands signals, and registers addresses and data information of the *k*-NN core, based on the signals received from the buffer controller, so that the neuro core can operate. Additionally, it receives the 16-bit data and ready signal from the neuro core and delivers the sampled data to the buffer controller.

#### 3.2.2. Neuro Core

The neuro core consists of an instruction decoder, a memory cell controller, and a classifier. The memory cell controller includes 1024 memory cells, each capable of storing 128 byte-sized training data points, in order to perform the *k*-NN algorithm. To undertake learning and inference in the neuro core, it is necessary to interpret the command signals and register address signals received from the neuro controller. This operation is performed by the instruction decoder, which then transfers the command information and data of the memory cells to the scheduler within the memory cell’s controller module. The scheduler receives command information and data and schedules the operation of memory cells. Additionally, it outputs a finish signal to notify the instruction decoder that it is ready to receive the next command, when the learning is completed.

## 4. System Flow

### 4.1. Entire System Flow

**Figure 5:**
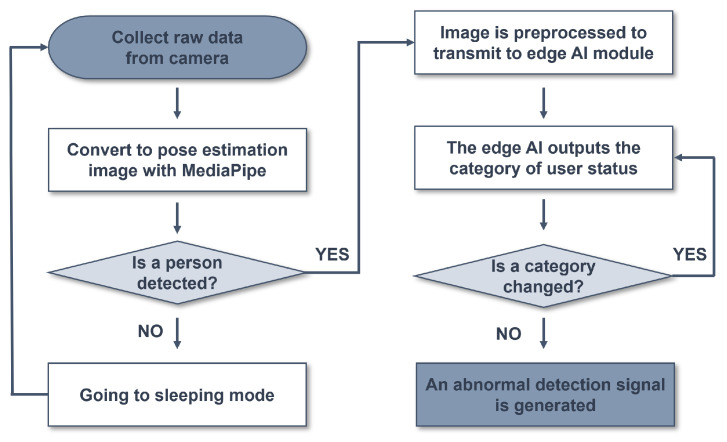
Flowchart of the intelligent monitoring system.

The flow of the intelligent monitoring system is shown in Figure 5. First, raw data are collected from the camera. The collected raw data go through the MediaPipe on MCU no. 1 to detect the presence of a person. If no person is detected in this step, the wake-up function of the system is activated to switch to sleeping mode. If a person is detected, preprocessing of the image is performed before it is transmitted as input data to the edge AI module on MCU no. 1. The preprocessed image is then sent to the edge AI module, which outputs a category representing the user’s state. This category is transmitted to MCU no. 2, which generates an abnormal detection signal if there is no change in the category for more than 30 h, indicating that there is a problem with the elderly individual living alone.

#### 4.1.1. Flow of Image Preprocessing

**Figure 6:**
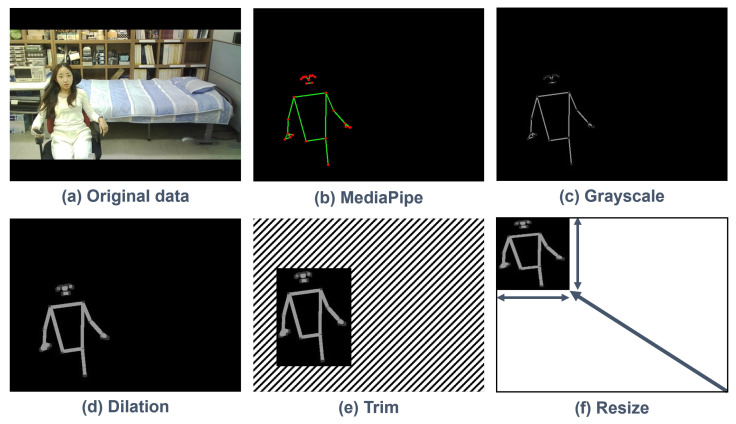
Flow of image preprocessing.

Figure 6 shows the overall flow of image preprocessing that occurs through the algorithm of the intelligent monitoring system. The image preprocessing is performed in MCU no. 1. The raw image data acquired from the camera module convert to an image with pose estimation through MediaPipe and go through dilation computation via grayscale conversion. The region representing the pose of a person is trimmed and resized according to the vector size of the N-cell. The resized image data are flattened to vector data in one dimension to transmit to the edge AI module.

#### 4.1.2. MediaPipe

MediaPipe is an open-source framework developed by Google that provides real-time processing and machine learning for video and audio data. The MediaPipe includes various algorithms used in computer vision and machine learning fields. Moreover, 3D pose estimation is a computer vision technique that captures human motion and estimates it in the form of a skeletal structure. To track poses, MediaPipe extracts 2D images from camera or video inputs. Next, using 33 3D landmarks, MediaPipe detects the joints and body parts of a person from the 2D image and then converts them into a 3D model. In this process, deep learning is utilized to handle the detected 2D joint data and estimate the 3D pose, which allows for the real-time tracking of a person’s movements. MediaPipe’s pose tracking is even more convenient to use because it works without markers. Moreover, this technology is also available on mobile devices, allowing for real-time motion capture functionality to be implemented.

### 4.2. Edge AI Module Algorithm

**Figure 7:**
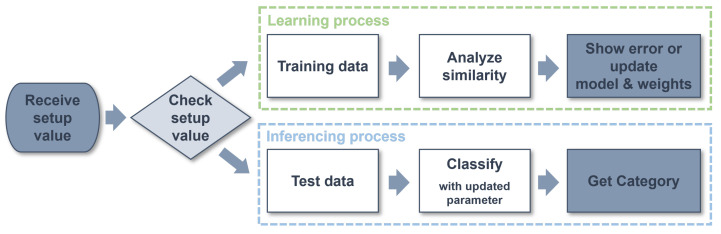
Flowchart of the edge AI module.

Figure 7 shows the proposed flow of the edge AI module. First, the setup data are received to determine the operation of the edge AI module for learning and inference. Next, depending on the values of the setup data, the incoming data are distinguished as either data for learning or data for inference, and the corresponding actions are performed. If the input data are identified as learning data, the 128 byte vector data points and the category values that distinguish the data are received together. The received training data are analyzed for similarity with the existing data with the same categories that have been trained and then saved as new training data or output errors. If the data are identified as data for inference, the edge AI module calculates the distance between the trained data in parallel. Distances from the training data are calculated in parallel, ensuring that the system operates without performance degradation, even as the training data volume increases. Afterward, the edge AI module classifies the closest training data with the smallest distance and outputs the category values of the data as the inference results. The Manhattan distance measurement method was applied to calculate the distance between test data and training data. When implemented in hardware, the Manhattan distance offers advantages in terms of reduced area and power consumption. This is because it can calculate distance without using multiplication units that occupy a large area.

## 5. Experiment

### 5.1. Software Simulation

**Figure 8:**
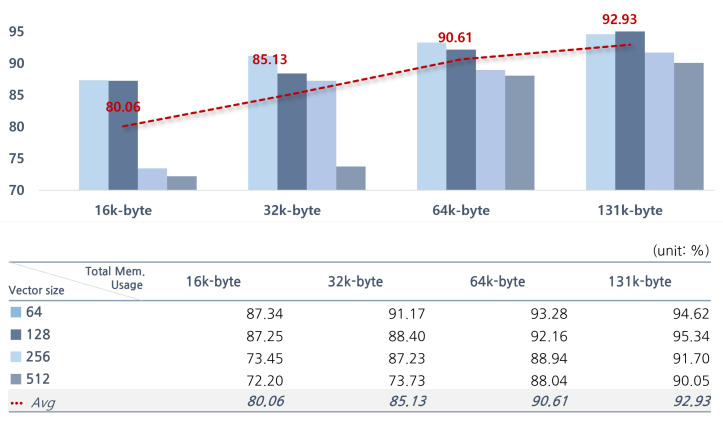
Accuracy of each specification.

The accuracy of the proposed edge AI module varies according to its specifications. The configurable parameters are the number of N-cells and the vector size. Therefore, we used a Python-based software simulator to determine the optimized specifications before implementing the edge AI module as hardware. The software simulator models the operation of the edge AI module and performs the *k*-NN algorithm based on the Manhattan distance [38]. It provides parameters for setting the number of cell modules and memory sizes within the accelerator. Therefore, the inference performance can be simulated according to the specifications of the edge AI module. In this paper, to analyze the accuracy of an intelligent monitoring system for elderly people living alone, we measured the accuracy based on image data input through a camera. The system performs the classification of ADL for humans. The data are divided into three labels: sitting, standing, and lying; the dataset includes a total of 6144 frames. Considering that the system is implemented as an embedded system, the maximum memory usage was limited to 131 k-bytes, and the accuracy was measured according to the number of memory cells and the data size. Figure 8 shows the accuracy according to the total memory usage. The accuracy was calculated for training datasets by measuring the rate of correct predictions for the three categories (lie, stand, sit) using Equation (1). In Equation (1), “true positive (TP)” represents the number of cases where the model correctly predicts the true category. The “Total train data number” corresponds to the number of memory cells used in the study.

(1) $$Accuracy\left(\%\right)=\frac{TP_{lie}+TP_{stand}+TP_{sit}}{Total\;train\;data\;number}\times 100$$

**Figure 9:**
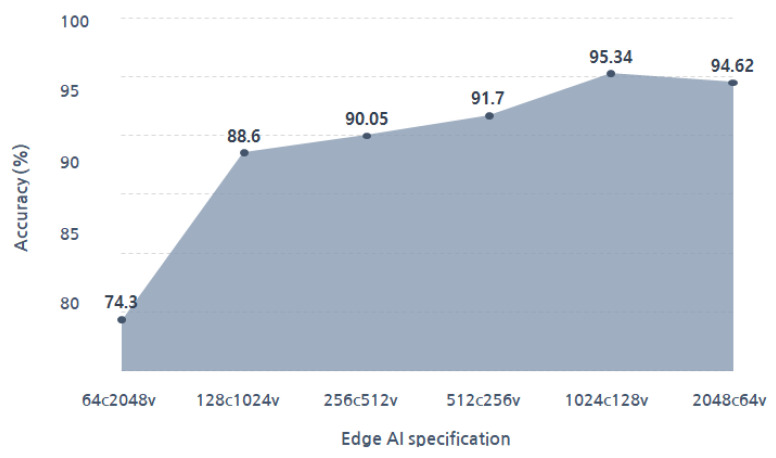
Accuracy of various parameter configurations under 131 k-bytes of total memory usage.

Also, accuracy was measured for four cases of total memory usage: 16 k-bytes, 32 k-bytes, 64 k-bytes, and 131 k-bytes. For each memory usage, we divided them into 4 cases with vector sizes of 64 bytes, 128 bytes, 256 bytes, and 512 bytes, respectively, resulting in a total of 16 cases. On average, the accuracies, according to the memory size of each case, were 80.06% at 16 k-bytes, 85.13% at 32 k-bytes, 90.61% at 64 k-bytes, and 92.93% at 131 k-bytes. The highest accuracy was achieved at 131 k-bytes. To decide the optimal parameter configuration, additional experiments were conducted at a memory usage of 131 k-bytes. In Figure 9, the accuracies of various parameter configurations under 131 k-bytes of total memory usage are presented. The configuration with 1024 memory cells and had a memory size of 128 bytes (1024C.128B) had the highest accuracy at 95.34%. We confirmed it as the most optimized model for the proposed system.

### 5.2. Hardware Implementation

**Figure 10:**
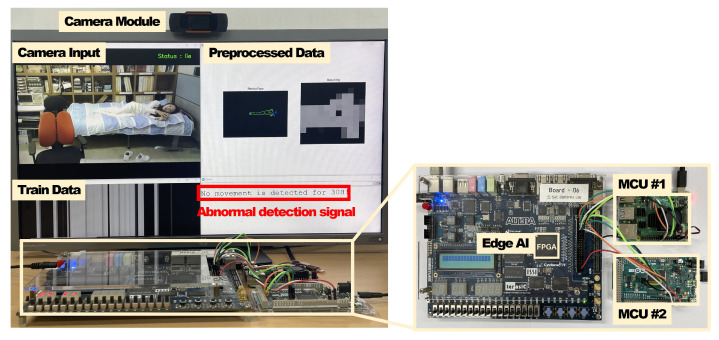
Hardware implementation environment.

Figure 10 presents the implementation and verification environment for validating the operation of an intelligent monitoring system. The intelligent monitoring device consists of two MCUs, a camera module, and a DE-2 board. The DE-2 board includes an edge AI module and an SPI module for external communication. The edge AI module and SPI modules were designed utilizing Verilog HDL and implemented with FPGA prototyping. The camera module is connected to MCU no. 1, and the two MCUs are connected to the general-purpose input/output (GPIO) of the DE-2 board, which contains the edge AI; they communicate with each other through the SPI protocol. The implemented edge AI module learned a total of 1024 training data points, with both the training and inference data sized at 128 bytes. This is the configuration that had the best performance in the software simulation and was adopted as the most suitable model for the proposed system. The proposed system conducted experiments on image data where people appeared and left repeatedly in an environment modeled after the living environment of an elderly individual living alone. The image data consist of a total of 6144 frames, including everyday actions, such as lying on a bed, sitting in a chair, and standing up. We verified that the proposed system correctly triggers an abnormal detection signal from MCU no. 2 (connected to the server) when a person is detected but remains motionless for over 30 h.

When an edge AI is implemented in hardware, the increase in memory size and computational capabilities result in a trade-off between the design area and performance. The typical design goal of an edge AI is to achieve optimal performance and minimal area at the same time. The edge AI includes dedicated memory for storing training data in memory cells, and the available training data numbers are constrained by the number of memory cells. Therefore, the numbers and sizes of memory cells are adjusted appropriately to design an edge AI that has the best performance in a limited area. To achieve this, it is important to simulate under various situations to find the optimal design. The total memory usage of the proposed edge AI is defined as the product of the number of embedded memory cells and the memory size contained in each memory cell. Even when implemented in hardware, the result is the same as in Figure 9, which is the result of the software simulation. It shows the accuracy of a monitoring system based on the number and size of memory cells, given that the total memory usage of an edge AI is 131 k-bytes. Also, it illustrates that there is a difference in the performance, depending on the specifications, despite the memory usage being the same. Furthermore, even if the total memory usage is identical, there may be differences in the design area of the edge AI module, depending on its specifications. Table 2 shows the FPGA resource utilization of the edge AI module, which has a memory usage of 131 k-bytes, based on its specifications. These results were synthesized in a 180 nm CMOS technology environment. As the number of memory cells increases, the number of computational units for distance operations and the sizes of the combination circuits for comparing distance values also increase, leading to an escalation in resource usage for the memory cell controller and classifier. When using 2048 memory cells of 64 bytes each (2048C.64B), it occupies 26 times more resources compared to applying 64 memory cells with a size of 2048 bytes (64C.2048B). Therefore, to determine the specifications of an edge AI according to the application, it is necessary to consider the trade-off between the design area and accuracy based on the number of memory cells and memory size. In this study, taking into account this trade-off, we selected an edge AI with 1024 cells and a memory size of 128 bytes for our specifications. The power consumption of the selected edge AI module is shown in Table 3. This metric was taken under the same conditions as hardware resource usage measurements, and the total power consumption was 344.44 mw. The power consumption of the classifier was 198.742 mw, accounting for 57.7% of the total power consumption. The memory cell controller followed, consuming 145.501 mw or 42.2%, and the instruction decoder was 0.0983mw, which was less than 1%.

## 6. Conclusions

In this paper, we proposed an intelligent monitoring system with privacy preservation based on edge AI. The proposed system employed an edge AI module, a camera module, and two MCUs that identified the state of an elderly person living alone, and detected the possibility of a lonely death. The system employed the edge AI module to block the data transmission between MCU no. 1 and MCU no. 2 in order to ensure reliability and prevent privacy leakage. Additionally, the system used MediaPipe, image preprocessing, and AI technology to operate the intelligent monitoring system in any environment. Prior to hardware implementation, a software simulation was conducted to determine the optimal specifications for edge AI. The simulation yielded an accuracy of 95.3% with 1024 cells and 128 bytes. The optimized edge AI was then implemented on an FPGA, which achieved the same accuracy as the hardware experimental settings. When synthesized on a 180 nm process, the accelerator had 1,516,046 NAND gates and consumed 344.44 mW. The system’s feasibility was demonstrated through experiments with 6144 frames, which confirmed that the system correctly generated abnormal detection signals when a person was present but did not move for a certain period of time.

However, we encountered several limitations in our research. First, we were unable to conduct experiments with elderly individuals in their real living environments. The proposed system using the board and MCU was designed for experimental purposes and was not packaged as a fully commercial product. Our primary focus during the initial development phase was on functionality and performance validation. To package it as a final commercial product and design it to fit real living environments, additional development and testing would be required. Although we conducted experiments in an environment that closely simulated the living conditions of solitary elderly individuals, there were some differences between the laboratory setting and real living environments. Residential environments can have complex layouts and various lighting conditions, among other factors, which could potentially impact the system’s operation in real living environments. Therefore, the results obtained in the laboratory may not fully reflect certain environmental factors or provide a complete representation of the system’s performance in a real living environment. However, we faced constraints in terms of cost and time. Developing a system for deployment in residential environments, ensuring the perfect packaging of the board and MCU, data collection and analysis, and cooperating with experimental subjects all required significant resources and effort. One future research direction will be to focus on experiments and improvements, especially in real-life environments.

Secondly, we conducted experiments on only three specific movements. We chose to classify only three simple movements (sitting, lying down, and standing up) for several reasons. Firstly, elderly individuals may have difficulty performing complex and challenging movements due to limited mobility. Therefore, focusing on classifying these three essential movements was a practical consideration for real-life scenarios. Additionally, adding numerous movements would increase the complexity of the recognition model and subsequently require more memory usage. Monitoring systems for elderly individuals are typically integrated into small-scale embedded systems with limited memory capacity. By training the model only on essential movements necessary for daily living, we reduced the required amount of data, computational complexity, and minimized memory usage.

Thirdly, we implemented a low-power design, but there is a lack of information regarding power consumption measurements. It is challenging to measure the power consumption of MCU nos. 1/2 and the artificial intelligence processor separately. However, we generally believe that having a power-saving mode, such as the wake-up function, enables more efficient battery usage. The wake-up function implemented in MCU no. 1 confirms the presence of a person, and subsequent computation processes proceed only when a person is detected. Therefore, power is consumed only during the preprocessing stage in MCU no. 1 when the presence of a person is confirmed. The power-saving mode reduces the power consumption when there is no user input, leading to energy savings. On the other hand, without a wake-up function, the system remains active, resulting in the continuous power consumption in MCU no. 1, the artificial intelligence processor, and MCU no. 2, even if a person is not detected. As a result, it is natural for power consumption to be relatively higher when a wake-up function is not implemented.

In future work, we plan to create an onboard monitoring system device and deploy it in real residential environments for experimentation and improvement. Additionally, we will develop image processing algorithms using a fish eye lens to enable the monitoring of large areas and further advance the single sensor-based monitoring system.

## Figures and Tables

**Table 2:** The hardware resource usage for the edge AI module is measured using the 2in1 NAND gate count.

Spec	Inst. Dec.	Mem. Cell Controller	Classifier	Neuro Core	Edge AI Module
2048c64v	322	1,162,841	1,778,715	2,941,878	2,943,295
1024c128v	361	604,474	909,794	1,514,629	1,516,046
512c256v	371	313,766	475,098	789,235	790,652
256c512v	342	162,422	247,087	409,851	411,268
128c1024v	371	83,936	128,168	212,475	213,893
64c2048v	342	43,432	67,549	111,323	112,741

**Table 3:** Power consumption of an edge AI module.

Spec	Inst. Dec.	Mem. Cell Controller	Classifier	Neuro Core	Edge AI Module
1024c128v	0.0983 mW	145.501 mW	198.742 mW	344.3413 mW	344.44 mW

## Data Availability

Not applicable.

## Abbreviations

The following abbreviations are used in this manuscript:

AI	artificial intelligence
FPGA	field-programmable gate array
IoT	Internet of Things
DB	database
k-NN	k-nearest neighbor
AR	activity recognition
ADL	activities of daily living
PIR	passive infrared sensor
SURF	speed-up robust features
MFCC	Mel-frequency cepstral coefficient
SVM	support vector machine
DCNN	deep convolutional neural networks
DNN	deep neural network
RRAM	resistive RAM
BNN	binarized neural network
MCU	microcontroller unit
SPI	serial peripheral interface
GPIO	general-purpose input/output
